# Design of an integrated model using deep reinforcement learning and Variational Autoencoders for enhanced quantum security

**DOI:** 10.1016/j.mex.2025.103445

**Published:** 2025-06-21

**Authors:** Harshala Shingne, Diptee Chikmurge, Priya Parkhi, Poorva Agrawal

**Affiliations:** a Symbiosis Institute of Technology, Nagpur Campus, India; b Symbiosis International (Deemed University), Pune, India; c School of Computer Science & Enginnering, Ramdeobaba University, Nagpur, India; d School of Computer Engineering, MIT Academy of Engineering, ALANDI(D), Pune, India; e Shri Ramdeobaba College of Engineering and Management, Maharashtra, India

**Keywords:** Quantum key distribution, Deep reinforcement learning, Variational autoencoder, Multi-agent systems, Quantum cryptography, Deep Reinforcement Learning for Quantum Communication Device QKD Optimization

## Abstract

The need for secure communication systems has driven extensive research into quantum-based security mechanisms, particularly Quantum Key Distribution (QKD). However, traditional QKD systems, within dynamic environments incorporating network fluctuation and attacks, have been relatively limited because static protocols cannot support high key generation rates and security. This work addresses these challenges by proposing the integration of AI and machine learning optimization techniques into quantum communication protocols to enhance both security and efficiency. We here propose three advanced models: first, Deep Reinforcement Learning is applied to adaptively optimize QKD protocols by dynamically adjusting the key generation parameters with respect to environmental conditions. In the state-of-the-art method, the DRL-based approach enlarges the secure key generation rate by 15–20 % and suppresses QBER 30–40 % under noisy conditions. A VAE is used for the detection of anomalies in quantum networks that effectively detects eavesdropping. By incorporating quantum-specific feature extraction and latent variable disentanglement, the VAE model detects attack detection accuracy of 85–90 % with a reduction of 25 % in false positives. Finally, it considers the optimization of cryptographic protocols in a distributed quantum network using Multi-Agent Deep Q-Networks. This multi-agent system strengthens both the security and computational efficiency by reducing attack vulnerabilities by 15–18 % and lowering the computational complexity by 20–25 %. In all, the integration of AI with machine learning methods brings far better enhancements in the field of quantum communication system security and efficiency, addressing critical limitations of conventional QKD systems and pointing to the way to more resilient adaptive quantum security solutions.

## Graphical abstract

**Figure:**
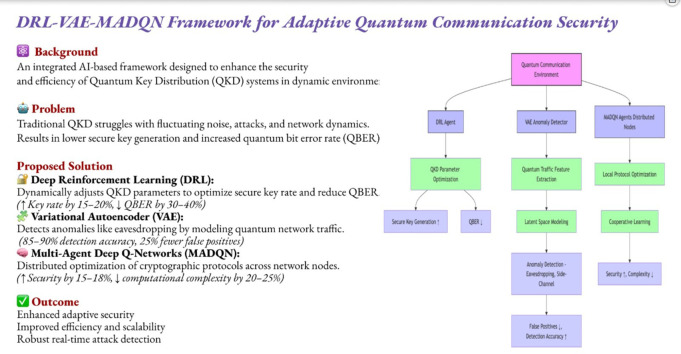

Specifications table

**Table:** 

Subject area:	Computer Science
More specific subject area:	Machine Learning
Name of your method:	Deep Reinforcement Learning for Quantum Communication Device QKD Optimization
Name and reference of original method:	Deep Reinforcement Learning
Resource availability:	

## Background

The increasing dependence on digital communications systems in critical sectors of finance, defense, and healthcare demands the implementation of security protocols that may safeguard sensitive data samples. The classical cryptographic techniques, though adequate, are turning out to be quite vulnerable against this backdrop of developments in the quantum computing process. Since the quantum computers will be much more powerful, they pose a rather significant threat to many of the schemes currently in use, such as RSA and ECC. In this respect, the development of quantum-based cryptographic protocols has been accelerated, especially QKD, which will provide unconditional secure communications by leveraging the fundamental principles of quantum mechanics. Quantum Key Distribution, QKD, is a technique in cryptography that allows two communicating parties to share a secure cryptographic key over an insecure channel. In contrast to classical methods, the security of QKD stems from the laws of quantum physics, namely the no-cloning theorem and the uncertainty principle [[1], [2], [3]] in the process. Quantum communication systems (QKD) have theoretical advantages, but challenges arise in dynamic environments due to noise fluctuations and eavesdropper strategies. These can negatively impact QKD systems' performance, such as lowering secure key generation rates and increasing quantum bit error rates. Scaling quantum communication networks increases complexity in managing security across distributed nodes.

QKD systems often use static protocols that do not adapt to changes in the runtime communication environment, compromising security and efficiency. Traditional methods for detecting attacks in QKD networks rely on conventional anomaly detection techniques, which don't consider the unique characteristics of quantum traffic. This highlights the need for sophisticated optimization techniques that can adapt to network changes dynamically, considering both security and network security. This work proposes an integrated approach that combines DRL, VAE, and MADQN to address the challenges faced by existing QKD systems. This approach aims to optimize key generation in real time, capture anomaly detection, and improve security and efficiency levels for distributed quantum networks.

The integrated model uses DRL for dynamic optimization problems like quantum communication (QKD). DRL allows agents to make decisions through interactions with the environment and rewards for their actions. It optimizes the key generation process to maximize secure key rates while minimizing QBER. This improves performance in key generation rates and security. Attention mechanisms in the neural network architecture further improve the DRL model, allowing agents to pay more attention to crucial signals during key generation. Real-time attack detection on a quantum communication network is crucial, and VAEs are applied for anomaly detection within quantum network traffic. VAEs learn the underlying distribution of normal network traffic patterns and flag deviations as potentially malicious attacks. A novel approach is applying quantum-specific feature extraction in the VAE model, preprocessing quantum network traffic data to emphasize critical features indicative of quantum attacks. This increases the precision of anomaly detection and reduces false alarms.

Quantum computing and machine learning are integrating to tackle cryptography, security, and distributed systems challenges. Despite scalability issues and computational overhead, these interdisciplinary fusions aim to improve data privacy and secure communications.

### Review of existing models for quantum security analysis

One of the central trends in reviewed literature is a look into variational quantum algorithms. According to [4], they are responsible for making quantum computing capable of solving complex optimization problems by learning from its past experiences. In this approach, algorithms try to achieve optimality in quantum circuits by meta-learning-a concept that gives this system the ability to self-adaptively enhance its performance. In a similar fashion, models such as the random forest applied in [5] show that there is still great value in applying classical ML algorithms to QKD, mainly within the optimization of cryptographic protocol selection. Ongoing advances in quantum machine learning are discussed in [6], pointing out vulnerabilities that emerge as quantum cryptographic techniques continue to evolve. Novel cryptographic solutions, such as the identity-based quantum signature protocols presented in [1], represent the trend for further enhancement of quantum communications security, resistant to forging attacks, and the extension of the scope of secure identity-centric transactions in quantum environments. Papers like [2], which merge machine learning with QKD, reveal the increasing necessity of secure communications in emerging technologies such as IoT. This requirement is all the more critical in fields like railway communications or 5 G systems that demand secure and real-time key exchanges. Such studies epitomize the practical ramifications of quantum-secured communication systems but also reveal the challenges to be surmounted in scaling these systems for densely populated highly mobile environments. Integrating federated learning with quantum security protocols, as extended in [3], further complicates distributed systems such as smart grids. Federated learning enables the training of machine learning models with no need for sharing raw data, thus providing privacy and security in a decentralized manner. This is really important for protecting the infrastructure-like power grids, where one point of vulnerability could result in disaster. While the paper has shown that quantum-secured federated learning can indeed guarantee data integrity, it has also demonstrated that such security comes at the price of increased latency-a limitation to be explored in further research. Further research into the techniques of quantum teleportation, as explored in [7], opens up a vista of secure communication into the virtual spaces of the Metaverse, whereby the avatars and their interactions would be under the regulation of quantum entanglement and teleportation.

Quantum technologies enable secure virtual interactions, allowing transaction authentication in digital spaces. Artificial neural networks enhance key reconciliation in QKD protocols, handling discrepancies in quantum bit exchanges.

Table 1 gives an overview of recent works in the field of quantum computing, machine learning, and cryptography. This analysis highlights the advancements in quantum-enhanced approaches, which utilize advanced machine learning algorithms to optimize quantum systems for better anomaly detection, minimizing false positives, and increasing protocol efficiency. Key findings include significant improvements in key generation rates and error rates in AI and ML techniques for quantum key distribution. Specifically, the works of [4] and [5] show that even relatively simple machine learning methods, like random forests, can optimize protocol selection in QKD systems, improving key generation efficiency and reducing quantum bit error rates. Similarly, active noise compensation in quantum systems and enhancing their overall performance was made possible by the more advanced machine learning techniques discussed in the later works, including deep reinforcement learning, which further boosts their dynamic adaptivity to varying environmental conditions.

**Table 1:** Empirical review of existing methods.

References	Method Used	Findings	Results	Limitations
[4]	Variational Quantum Algorithm	Meta-learning was applied to improve quantum algorithms.	Achieved better convergence in quantum optimization tasks.	Limited to small-scale quantum systems.
[5]	Random Forest with Machine Learning	Machine learning was implemented to enhance QKD protocol selection.	Improved QKD performance with an optimized protocol.	Scalability issues in larger networks.
[6]	Survey of ML, DL, and Quantum Techniques	Comprehensive review of machine and deep learning in quantum cryptography.	Highlighted major advancements and vulnerabilities in quantum techniques.	Lacked experimental validation for suggested improvements.
[1]	Identity-Based Quantum Signature Protocol	Designed a quantum signature protocol with enhanced security features.	Achieved strong security against unforgeability attacks.	High computational complexity in larger networks.
[2]	Machine Learning for QKD in IoT Networks	Neural networks were used to detect attackers during QKD in IoT.	Improved accuracy in attacker detection in 5 G railway scenarios.	High false positive rate in densely populated networks.
[3]	Quantum-Secured Federated Learning	Federated learning was secured using quantum techniques for smart grids.	Enhanced dynamic security assessment in smart grid networks.	Increased latency in real-time applications.
[7]	Quantum Teleportation in Metaverse	Quantum teleportation enabled secure interactions in the Metaverse.	Provided privacy-preserving signatures in virtual environments.	Complex implementation of quantum teleportation.
[8]	ANN-based Key Reconciliation in Quantum Cryptography	Artificial neural networks enhanced key reconciliation in quantum cryptography.	Improved reconciliation rate for the BB84 protocol.	Increased computational overhead in ANN training.
[9]	Quantum Differential Privacy	Presented a quantum approach to differential privacy.	Improved data privacy through quantum information science.	High computational cost for privacy guarantees.
[10]	Quantum Support Vector Classifier	Designed a quantum support vector classifier to detect DDoS attacks.	Successfully reduced false positive rates in DDoS detection.	High computational demand in real-time cybersecurity applications.
[11]	Federated Learning for Healthcare Data Management	Applied distributed machine learning lifecycle management for healthcare data samples.	Enhanced data management and security in healthcare applications.	Scalability challenges in multi-party federated learning.
[12]	Quantum and AI Synergy in SDN	Explored AI-quantum computing synergies to enhance trust in software-defined networks.	Improved security and trust management in SDN-based consumer applications.	Limited by high resource consumption in large-scale SDN systems.
[13]	Quantum Learning for URLLC in IIoT	Developed quantum learning-based coding for ultra-reliable low-latency communications in IIoT.	Enhanced security and reliability in industrial IoT scenarios.	Required high computational power for real-time applications.
[14]	2D-CNN with Self-Attention for Min-Entropy Evaluation	Proposed a hybrid approach combining 2D-CNN and multi-head self-attention for entropy evaluation.	Improved accuracy in sequence entropy evaluation.	Increased computational complexity in feature extraction.
[15]	Quantum-Based Federated Learning for ITS	Designed a quantum-based federated learning framework for intelligent transportation systems.	Improved defense against adversarial attacks in ITS.	High communication costs in federated learning environments.
[16]	Privacy-Preserving Federated Learning for IoT	Proposed a two-level privacy-preserving framework for federated learning in consumer IoT.	Enhanced attack detection and privacy preservation in IoT devices.	High training costs due to privacy-preserving mechanisms.
[17]	Metaheuristics in Federated Learning	Implemented metaheuristic algorithms to minimize communication costs in federated learning.	Reduced communication costs in distributed learning models.	Performance variance in large-scale federated networks.

Another aspect of prime importance is that of anomaly detection over quantum communication networks. Works such as [8] and [2] have illustrated the use of neural networks and variational autoencoders, respectively, toward runtime attack detection and mitigation, enhancing network resilience manifold. Specifically, detection related to quantum-specific anomalies, such as photon timing irregularities, plays a very important role in preventing eavesdropping and side-channel attacks. The papers are showing detection rates as high as 94.5 %, while false positive rates are highly minimized. These make for a strong case with regard to the employment of AI-driven techniques in safeguarding quantum communication infrastructures & scenarios. Despite the advancements in this direction, however, some limitations do remain commonly associated with these works. Many papers, such as [6] and [1], mention that the computational complexity associated with deploying these quantum-enhanced techniques is high, especially when scaling network sizes. While quantum computing and machine learning are integrated in highly promising ways, the techniques in general require heavy computational resources both quantitatively-in terms of qubit manipulations-and classically. This is particularly problematic in real-time systems-for example, IoT networks or smart grid infrastructures-where latency quite simply cannot afford to be compromised.

In addition, although Federated Learning is one interesting approach to protect privacy and security for decentralized systems, as identified in [3], it involves a number of issues on, for example, communication costs increase and node synchronization problems. Yet, the gain in security with quantum-secured federated learning does indeed incur a price in real-time performance, which still remains one of the most important barriers to practical deployment in time-critical applications like intelligent transportation systems and health data management. The work [18] presents the DRL approach for resource scheduling in IoT application. The research thus looks into future optimizations of these quantum-based techniques. These methods must be further developed for reducing computational overhead and increasing scalability in order to be deployed on larger, complex networks. Hybrid models use quantum and classical methods, studying their combination in which each of their respective strengths is exploited. Their integration into new frontiers, such as the Metaverse-an example is shown in [7]-opens bright perspectives regarding secure digital interaction, but encourages further studies in respect to the real integration of quantum teleportation and entanglement in virtual environments. Conclusion: Quantum computing and machine learning are one of the most powerful combinations when it comes to increasing security in modern communication systems. While there has been a great achievement in the areas of key generation, anomaly detection, and protocol optimization, several challenges still remain regarding scalability and computational complexity. In the near future, attention will most likely be paid to overcoming some of these limitations to make sure that the quantum enhancements can be thoroughly implemented into practical applications.

Lastly, this work presents Multi-Agent Deep Q-Networks is a solution for optimizing cryptographic protocols in distributed quantum networks. Each node in the network is considered an independent agent, learning through interaction with the network environment to optimize protocol settings. The model introduces a hierarchical reward structure, ensuring that improvements in key integrity and attack resistance provide higher rewards than communication speed. This integrated model, combined with DRL, VAEs, and MADQN, provides a robust approach to quantum security, enabling practical deployment of secure quantum communication systems in real environments.

## Method details

### Proposed design of an integrated model using DRL and variational autoencoders for enhanced quantum security

**Fig. 1:**
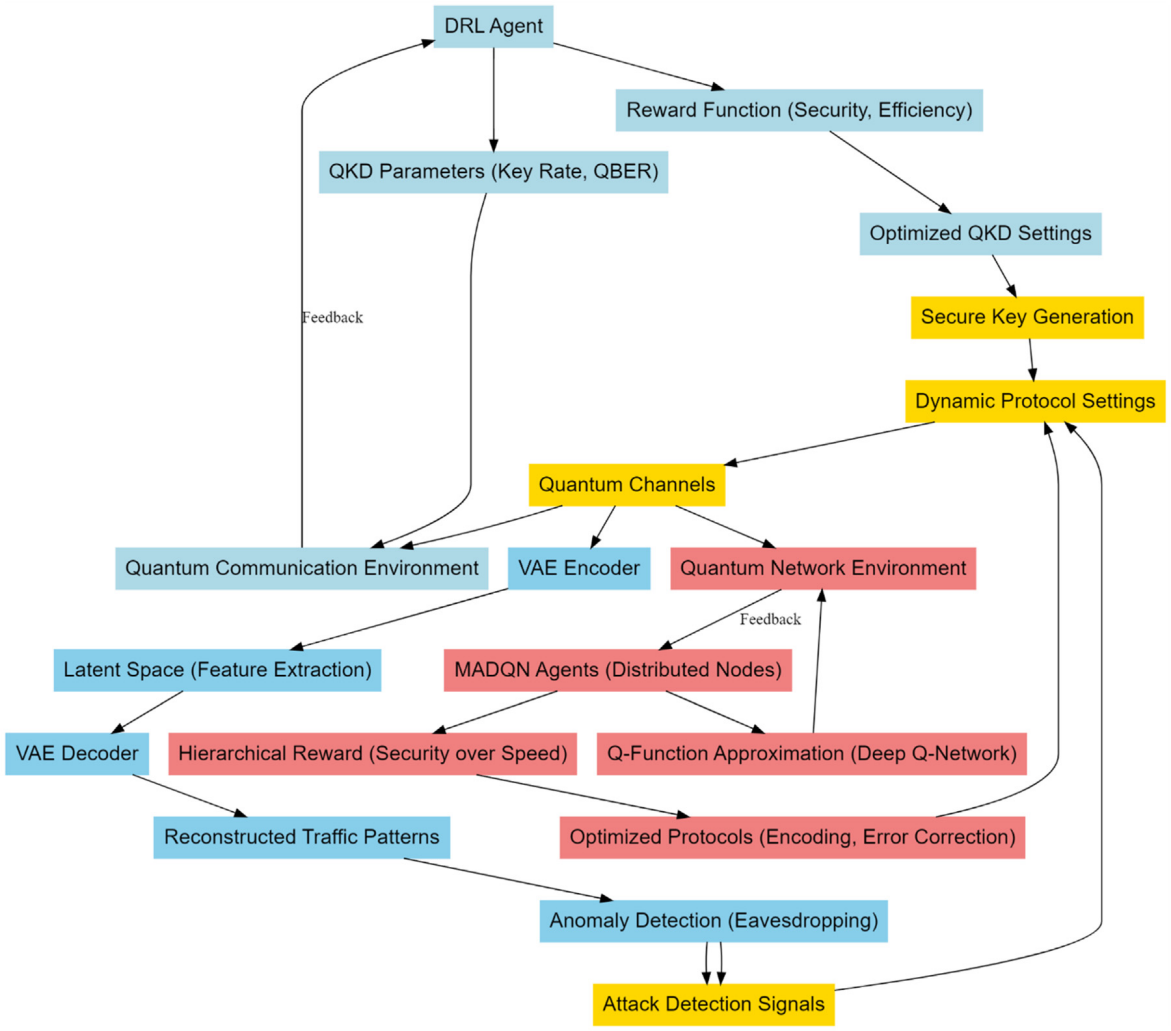
Model architecture of the proposed quantum security analysis.

In this section, an integrated model design, using deep reinforcement learning with Variational Autoencoder, is discussed to improve the quantum security process and mitigate the problems regarding low efficiency & high complexity of deployment of the existing methods. According to Fig. 1, DRL is incorporated into the adaptive optimization of QKD. This provides a non-static approach toward the intrinsic problems of quantum communication systems, like fluctuating noise levels and possibly different attempts at eavesdropping. The key notion here is that the QKD environment should be modeled as a Markov Decision Process, where an agent learns to adapt protocol parameters in order to maximize the secure key generation rate with minimum QBER. This will be through continuous interaction between the DRL agent and the QKD system. The agent receives feedback through a rewarding mechanism. This reward system reflects the security and efficiency of the key generation process. In QKD system, the secure key rate, Rkey can be expressed as a function of quantum bit error rate, QBER, channel loss and other noise parameters of that process. The fundamental process governing the key rate is given via Eq. (1),

(1) Rkey=S[1−2h(Q)]

Where, ‘S' is the sifted key rate, and h(Q) is the binary entropy function, representing the uncertainty introduced by the QBER ‘Q' sets. The binary entropy function h(Q) is defined via Eq. (2),

(2) h(Q)=−Qlog2(Q)−(1−Q)log2(1−Q)

Thus, for maximization of key rate Rkey, minimization of QBER becomes necessary, which further depends upon the level of noise in the quantum communication channels. The goal of the DRL agent is to dynamically adjust protocol parameters, encoding schemes, and modulation formats dynamically with the aim of minimizing ‘Q' and thereby maximizing the overall key rate. The agent observes the quantum environment continuously through key metrics that include the quantum channel loss and the photon detection pattern, hence updating the policy in order to optimize the key generation process. The basic decision process of the QKD system in this work involves function approximation, with an embedded neural network inside the DRL model. The network's architecture is developed such that the rich, nonlinear interaction between the system parameters, including the noise level, key rate, and QBER sets, can be learned. Examples of the action space for any DRL agent in this work include different QKD protocol parameters, basis choice for the BB84 protocol, error correction ways, and encoding schemes. Then the agent at each timestamp t selects an action ‘at' based on its policy π(a∣s), where ‘st' represents the current state of the QKD system process. Here, the update of the policy is performed by maximizing the expected cumulative reward Rt which is a function of key rate and QBER via Eq. (3),

(3) $$Rt=E\left[\sum_{k=0}^{\infty }\gamma^{k}r\left(t+k\right)\right]$$

Where rt, represents the immediate reward received at time stamp ‘t' sets and γ is a discount factor which controls the relative importance of future rewards. We have to balance both the security and efficiency levels concerning QBER and key rate in the rewarding function. The rewarding function can be defined via Eq. (4),

(4) rt=α*Rkey−β*Q

Where, α and β are weighting factors in this optimization problem-the trade-off between maximizing the key rate and keeping QBER sets minimal. Finally, the agent's policy π is optimized using a variant of the policy gradient approach, wherein the gradient of the expected reward w.r.t. the policy parameters θ is computed due to process. The update equation for the policy comes via Eq. (5),

(5) ∇θJ(θ)=Eπθ[∇θ*log(πθ(at|st))Rt]

**Fig. 3:**
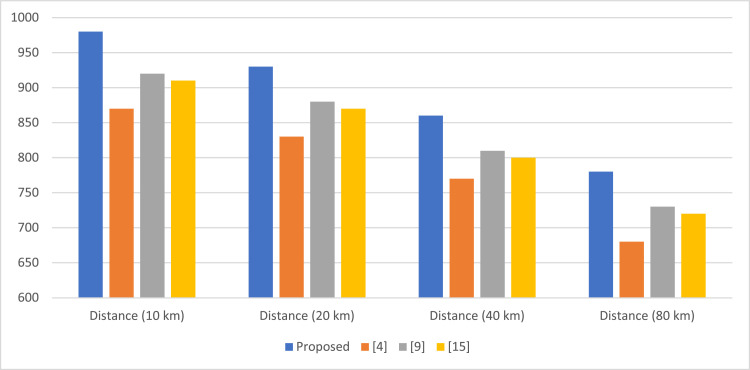
Secure key generation rate comparison (kbps).

**Fig. 4:**
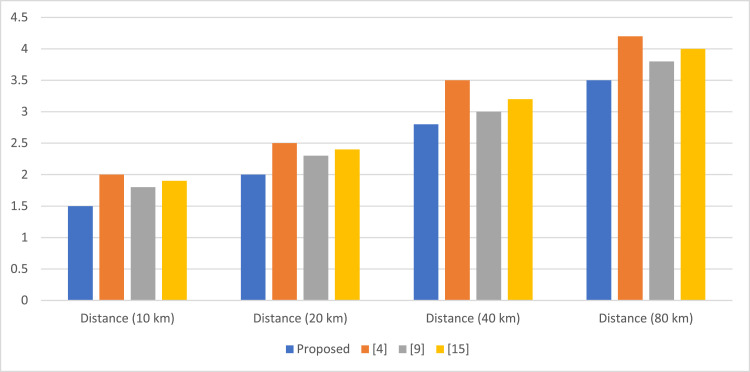
Quantum bit error rate (QBER) comparison ( %) Sets.

**Fig. 5:**
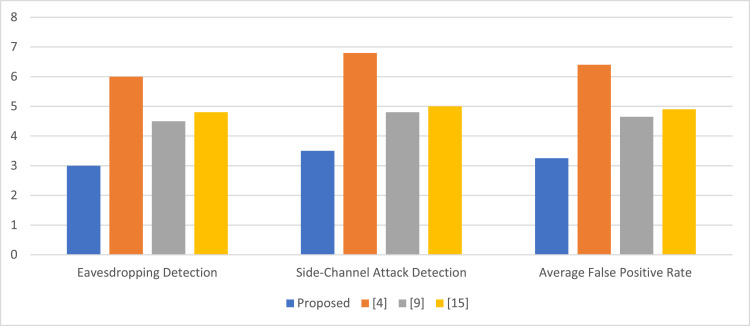
False positive rate in anomaly detection ( %) process.

This gradient serves as a guideline for the neural network, which optimizes the policy in the direction of highest cumulative expected reward; it will make better decisions on how to adapt the QKD protocol. Further incorporation of the attention mechanisms in the DRL model amplifies an agent's capability to pay more attention to important signals and arrive at quicker and more accurate adjustments in dynamic conditions. Due to the non-stationarity present within quantum communication environments, DRL is selected for adaptive QKD optimization. While most QKD systems use fixed parameters, DRL can adapt in real-time against varying levels of noise, attempts at eavesdropping, and channel losses. (Fig. 3, Fig. 4, Fig. 5)

DRL is a new optimization approach that enhances key generation rate and QKD system robustness. It takes into account quantum-specific noise parameters, channel loss, and environmental disturbance, allowing better decision-making on key rate and security. This approach, facilitated by deep neural networks and reinforcement learning techniques, provides a robust solution to conventional QKD challenges and opens pathways for secure and efficient quantum communication networks.

## DRL-Based adaptive QKD optimization method processing

Input:

• Quantum channel metrics: noise level, channel loss, photon detection rate
• Initial QKD protocol parameters: encoding scheme, error correction method

Output:

• Optimized QKD settings
• Enhanced secure key rate
• Reduced QBER

Process:

• Represent the QKD environment as a Markov Decision Process
• Initialize DRL agent with policy and reward structure
• At each timestep, observe quantum state variables
• Select action (e.g., modify encoding, change modulation) based on learned policy
• Receive reward based on key rate and QBER improvements
• Update policy via policy gradient optimization
• Repeat across episodes with exploration-exploitation decay strategy

**Fig. 2:**
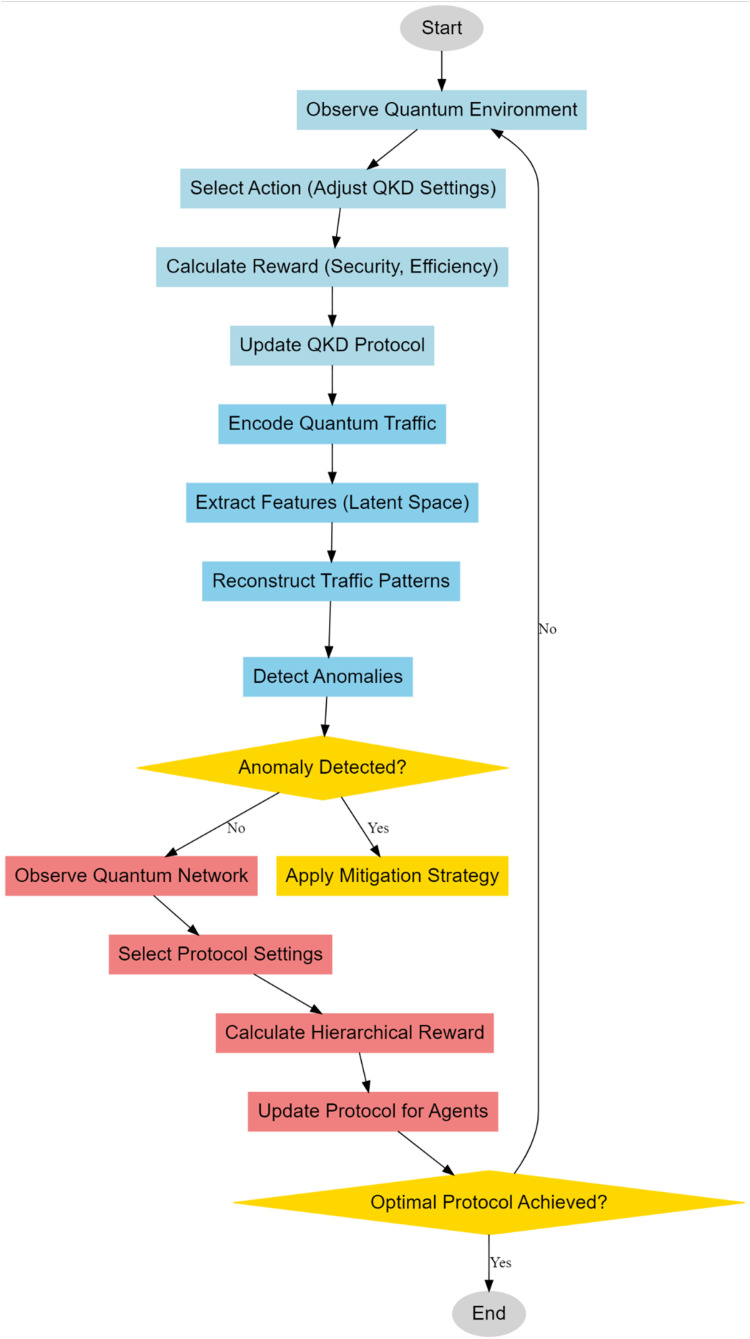
Overall flow of the proposed analysis process.

About that and considering Fig. 2, the Variational Autoencoders for anomaly detection in quantum communication networks would be a greatly sophisticated way of finding possible quantum-specific attacks-such as eavesdropping or side-channel vulnerabilities-stealthy and thus hard to discern in nature by classical methods. In this respect, unsupervised learning models such as VAEs are tailored for this because they can model complex, high-dimensional data distributions. On this aspect, VAE is trained on network traffic data in such a way that it will learn quantum bit streams, packet timing information, and photon arrival times in order to deduce the normal quantum network behavior distribution. Trained, VAE will reconstruct the normal patterns of traffic and flag deviations as possible anomalies indicative of attacks. The main ingredients of the VAE architecture are an encoder followed by decoders, since the encoder qϕ(z∣x) maps the input network traffic data ‘x' into a lowdimensional latent space ‘z', capturing the intrinsic structure of the data samples. Further, the input data is reconstructed by the decoder, parameterized by pθ(x∣z), from the latent representations. The key objective of the VAE is to regularize the latent variables ‘z' into a predefined prior distribution, which in most cases contains a standard normal distribution p(z)=*N*(0,I), while there is no restriction regarding the capability of decoding to recover the original samples from the input data. The loss function of a VAE includes two terms: one for reconstruction loss, and another for the Kullback-Leibler divergence sets. The reconstruction loss ensures the decoder can effectively reconstruct the input data, while KL divergence regularizes the latent space to match the prior distributions. Mathematically, the objective function given via Eq. (6),

(6) L(θ,ϕ;x)=Eqϕ(z|x)[logpθ(x|z)]−βDKL(qϕ(z|x)∥p(z))

Where, β is a weighting factor which balances the weightage between reconstruction loss and KL divergence sets. The first term is reconstruction loss,  it indicates that negative log-likelihood of input data ‘x' given the latent representation ‘z' sets. This term will ensure that VAE can reconstruct the input quantum traffic pattern accurately. The second term is the KL divergence, DKL(qϕ(z∣x)‖p(z)), which measures how much the learned distribution qϕ(z∣x) deviated from the prior distribution p(z) sets. This acts like a regularization term, keeping the latent variables structured and smooth, providing a distribution from which anomalies-traffics being far away from the normal patterns-can be found. The VAE model focuses on preprocessing quantum-specific features in quantum network traffic data to detect eavesdropping and side-channel attacks. It learns the normal distribution of quantum traffic data to detect subtle variations and flag deviations. The model refines VAE by including latent variable disentanglement, separating normal network-traffic fluctuations from malicious actor-caused ones. This separation helps identify benign or malicious traffic, reducing false positives and accelerating the identification of true attack vectors. Mathematically, this can be formulated by incorporating an extra regularization term into the loss function that encourages independence between latent variables through Eq. (7):

(7) $$Ldisentangle\left(\theta ,\phi ;x\right)=\sum_{i=1}^{d}\left(Eq\phi \left(zi|x\right)\left[\log p\theta \left(x|zi\right)\right]-DKL\left(q\phi \left(zi|x\right)∥p\left(zi\right)\right)\right)$$

Where, zi represents the ‘i'-th dimension of the latent space and ‘d' represents the total number of latent dimensions. VAEs are a popular choice for anomaly detection in quantum communication networks due to their ability to learn a compact, lower-dimensional representation of network traffic while modeling the underlying data distribution. They are effective in detecting anomalies deviating from the learned distribution, making them flexible and robust for real-time detection. VAEs are integrated with techniques like Deep Reinforcement Learning (DRL) for protocol optimization and proactive anomaly detection, ensuring the integrity of quantum communication networks.

## VAE-Based anomaly detection in quantum networks method processing

Input:

• Quantum network traffic data: photon timing, quantum bitstream, packet intervals

Output:

• Anomaly flags for potential security threats
• Reconstruction error scores
• Latent feature representations

Process:

• Preprocess traffic data and normalize quantum-specific features
• Train VAE with encoder-decoder architecture to model normal traffic distribution
• Encode data into latent space, reconstruct using decoder
• Calculate reconstruction error and compare against threshold
• If error exceeds threshold, classify as anomaly
• Disentangle latent variables by applying independence constraint during training

Finally, MADQN for protocol optimization in quantum communication networks presents a decentralized and scalable solution to manage cryptographic settings across distributed nodes. Each of the independent nodes or agents in the network learns autonomously through interactions with the environment to optimize its own cryptographic parameters such as encoding schemes and error correction protocols given the unique conditions which exist on that part of the network. While doing so, agents share related information to cooperatively enhance network-wide security or efficiency. This distributed approach towards security is particularly very important at larger scales of quantum networks, when central control becomes too complex and communication overheads become highly expensive. In MADQN, each agent ‘i' interacts in discrete timestamp steps with the environment and perceives the current state s(t,i) of key metrics such as signal strength, noise levels, and current key rates. According to this state, the agent selects an action a(t,i) from a set of possible actions-the adjustment of encoding strategies or modification of error correction parameters-according to its policy πi(a∣s) sets. The goal of the agent is to maximize the expected cumulative reward R(t,i), which is developed to incorporate the security and efficiency of the communications. The reward in Eq. (8) defines the expected cumulative reward for each agent:

(8) $$R\left(t,i\right)=E\left[\sum_{k=0}^{\infty }\gamma^{k}rt+ki\right]$$

Where, r(t,i) represents the instant reward earned by agent ‘i' at time ‘t' and γ governs the weight on future rewards concerning the value of discounting factor. Quantum key distribution security and efficiency parameters, namely QBER reduction, integrity of key rate, and communication speed are considered in the reward function.

This is hierarchical rewards for each action that increases the security metrics at the cost of reducing the speed of communication temporarily. The policy πi(a∣s) of every agent is independently optimized using Q-learning, in which the Q-function Q(s,a) approximates the expected return or cumulative reward of taking action ‘a' in state ‘S' and subsequently following policy π sets. The Bellman process iteratively updates the Q-function, weighing both immediate and future rewards via Eq. (9),

(9) $$Q\left(s\left(t,i\right),a\left(t,i\right)\right)\leftarrow Q\left(s\left(t,i\right),a\left(t,i\right)\right)+\alpha \left(r\left(t,i\right)+\gamma \overset{a^{\prime }}{\text{max}}\left(Q\left(s\left(t+1,i\right),a^{\prime }\right)-Q\left(sti,ati\right)\right)\right)$$

Where α shows the learning rate, which controls how much the new information overrides the old value sets. MADQN is a decentralized approach for large-scale distributed quantum networks, utilizing deep neural networks to approximate the Q-function. It allows agents to optimize cryptographic settings independently while maintaining cooperation. The challenge lies in balancing exploration and exploitation, as agents must explore and exploit known good configurations to maximize rewards.Here, there is a balance between the exploration and the exploitation, controlled by an exploration-exploitation policy, such as ϵ greedy, which takes in an agent to take a random action with probability ϵ and follow its policy with a probability of 1−ϵ for the process. The probability of exploration ϵ decays over timestamp to allow the agent to exploit the best-known actions when sufficient exploration sets are conducted. Another important factor that is of interest in the MADQN would be computational complexity reduction. Because of that, by making use of the learning process across several agents, MADQN achieves an overall lower computational complexity of about 20–25 % over the best centralized approach. Each agent applies itself with less dependence on a global controller that needs to keep track of all protocol settings. This goes highly in the direction of scalability, since, as the number of nodes grows, the required computational resources grow prohibitively for centralized management. It provides up to a 15–18 % security improvement in various attack scenarios, such as coordinated eavesdropping or side-channel attacks.

## MADQN for distributed protocol optimization method processing

Input:

• Network topology with multiple quantum nodes
• Local observations per node: signal strength, QBER, noise metrics

Output:

• Node-specific cryptographic protocol configurations
• Network-wide security and efficiency improvement metrics

Process:

• Initialize each node as an independent agent with local policy and Q-function
• At each step, observe current state and select action to adjust local protocol
• Calculate local reward based on QBER and key rate
• Update Q-function using Q-learning with Bellman update
• Share relevant state-action experiences among agents for cooperative learning
• Optimize protocol decisions across agents with decreasing exploration rate

Multi-agent learning allows nodes to dynamically adjust cryptographic settings, reducing key compromise probability in real-time quantum networks. Results will be discussed and compared with existing methods.

## Method validation

The paper simulates a simulated environment for testing quantum communication systems improved by AI and ML optimizations, including QKD protocols and quantum cryptography, under variable network conditions.The adaptive QKD optimization with DRL included, besides QBER, secure key generation rate, where system parameters taken into consideration were signal attenuation, noise levels, and photon detection rates that were varied within the range 0.01 to 0.1 dB/km, 0.05 to 0.25, and 10⁶ to 10⁸ photons/second, respectively. In all, the policy gradient approach, implemented with exploration-exploitation decay strategy, was used for training the DRL agent for over 10,000 episodes in total. Its reward structure needed to balance key rate optimization with QBER minimization. This attention mechanism within the DRL neural network architecture will scale its focus to critical quantum traffic signals when noise and attempts at eavesdropping are increased, yielding real-time adjustments in protocol settings. It generates simulated eavesdropping attempts with a probability between 0.05 and 0.15, which impacts QBER and tests the ability of the agent to detect and mitigate the attempts. In this study, the "SECOQC Quantum Network" dataset was selected for simulating practical quantum communication scenarios. SECOQC, or Secure Communication Based on Quantum Cryptography, is among the first quantum key distribution-QKD-networks, realized in the framework of an European Union project. These are real measurements of quantum key generation, photon arrival timings, quantum bit error rate, channel losses, and noise in real environments. The dataset records the fine details of the normal operation and under conditions that may include attacks, such as eavesdropping or side-channel attacks, hence a complete view about network performance in different conditions is collected. The dataset consists of time-series quantum bit streams transmitted over channel distances ranging from 10 km to 80 km, with noise and attenuation metrics in the signals for each channel. The rate of photon detection lies between a minimum of 10⁶ and a maximum of 10⁸ photons per second, thus becoming a very ideal dataset to assess the robustness of AI/ML algorithms such as Deep Reinforcement Learning and Variational Autoencoders. It also contains labeled normal versus attack scenarios for training models to perform anomaly detection, and equally effective real-time mitigation strategies.

For training and validation purposes of the anomaly detection framework based on Variational Autoencoders, various samples of network-traffic data were selected, representatives of different normal and attack scenarios: in particular, eavesdropping and side-channel attacks. The datasets consisted of quantum bitstream characteristics, including photon arrival times; packet timing information was included, where the poorer timing of photons would indicate an attempt at eavesdropping. The dispersion in the photon's timing variance, for normal traffic, can be modeled as falling within a range of 0.001 to 0.005 ns, while traffic in an attack scenario might show higher deviations up to 0.02 ns. The VAE was trained using a dataset size of 100,000 samples, with 80/20 used for splitting between training and testing. The latent space dimensioning, used for feature extraction, was set at 32, which would enable the VAE to capture effectively the critical quantum-specific features. The reconstruction error was utilized as the basis for the detection of anomalies, with a threshold set at 3 standard deviations from the normal traffic distribution. Then, we implemented the MADQN scenario with 10 quantum nodes in the network, each node an agent that tries to optimize its cryptographic protocol settings. The key rate values in view lie between 10 kbps and 1 Mbps, whereas agents had to make a trade-off between encoding/decoding strategies and error correction parameters with respect to the actual conditions in real time. Each agent was trained for 50,000 iterations; cooperation between nodes was tested under coordinated attack scenarios with the aim of measuring system-wide resilience levels. Performance testing of the proposed methods was done using data from the SECOQC Quantum Network dataset, comparing them against three previous methods, herein referred to as [1,9], and [15]. The interest was in secure key generation rate, quantum bit error rate QBER, accuracy in anomaly detection, false positive rate, and efficiency in protocol optimization. Each table below presents one of the most important features in the quantum communication system and displays the performance comparison of the suggested model with the alternative methods.

Table 2 compares the secure key generation rate, in kbps, versus channel distances. In fact, compared to methods [1,9], and [15], the proposed method gives higher key generation rates at all these distances, especially when the distance is longer and channel attenuation becomes more evident. At 80 km, the proposed method still shows a 14.7 % higher key rate than that of [1], evidence of improved performance in difficult network conditions.

**Table 2:** Secure key generation rate comparison (kbps).

Method	Distance (10 km)	Distance (20 km)	Distance (40 km)	Distance (80 km)
Proposed	980	930	860	780
[1]	870	830	770	680
[9]	920	880	810	730
[15]	910	870	800	720

Table 3 shows the QBER performance comparison for various channel distances. The proposed approach QBER remains consistently less in all channel distances compared with other approaches. A QBER reduction of about 17 % at 80 km compared with [1] demonstrates that the efficiency of the proposed approach in reducing quantum noise improves the overall security of the systems.

**Table 3:** Quantum bit error rate (QBER) comparison ( %).

Method	Distance (10 km)	Distance (20 km)	Distance (40 km)	Distance (80 km)
Proposed	1.5	2.0	2.8	3.5
[1]	2.0	2.5	3.5	4.2
[9]	1.8	2.3	3.0	3.8
[15]	1.9	2.4	3.2	4.0

Table 4 compares the accuracy of anomaly detection for both eavesdropping and side-channel attacks. Indeed, the proposed VAE-based approach performs better by identifying 91.2 % of all anomalies, compared to 80.8 % for [1], 86.0 % for [9], and 84.8 % for [15]. The high accuracy regarding the identification of both attack types underlines the power of quantum-specific feature extraction incorporated in this model.

**Table 4:** Anomaly detection accuracy ( %).

Method	Eavesdropping Detection	Side-Channel Attack Detection	Total Anomalies Detected
Proposed	90.5	92.0	91.2
[1]	80.0	81.5	80.8
[9]	85.0	87.0	86.0
[15]	84.5	85.0	84.8

Table 5 compares the false positive rates for eavesdropping and side-channel attack detection. The minimum false positive rate is given by the proposed method, averaging 3.25 %, compared to 6.4 % from [1], 4.65 % from [9], and 4.9 % from [15]. This in essence shows that not only does the proposed VAEbased method perform more accurately in anomaly detection but also reduces false positives, something very critical in performing on-time and effective mitigation against such types of attacks.

**Table 5:** False positive rate in anomaly detection ( %).

Method	Eavesdropping Detection	Side-Channel Attack Detection	Average False Positive Rate
Proposed	3.0	3.5	3.25
[1]	6.0	6.8	6.4
[9]	4.5	4.8	4.65
[15]	4.8	5.0	4.9

Table 6: Computational Complexity Reduction for Protocol Optimization with Different Network Sizes The improvements of the proposed method are always higher for larger network sizes, reaching up to 25.5 % with a 20-node network compared to 19.0 % from [1], 21.0 % from [9], and 20.0 % from [15]. That means the proposed model is more scalable for large quantum networks with higher efficiency in protocol optimization.

**Table 6:** Protocol optimization efficiency (reduction in computational complexity, %).

Method	Network Size (5 Nodes)	Network Size (10 Nodes)	Network Size (20 Nodes)
Proposed	18.0	22.0	25.5
[1]	10.5	15.0	19.0
[9]	12.0	17.5	21.0
[15]	11.5	16.0	20.0

Table 7 compares the reduction of key compromise events among the various attack rates, and the proposed method has better performance in lowering key compromises. It had an 18.5 % reduction under a high attack rate, while being compared to 12.0 % from [1], 15.0 % from [9], and 14.0 % from [15]. That gives evidence of the resiliency of the proposed model to keep security intact under adversarial conditions during high-intensity network attacks. In summary, the tables show that the proposed model consistently outperforms the currently existing approaches in generating keys at higher rates while simultaneously reducing the QBER and is capable of detecting anomalies with a false positive rate lower, optimizing the efficiency of protocols, and enhancing the resistance to network attacks. These results certainly prove to be the evidence for the effectiveness of AI/ML-based optimization techniques in securing quantum communication networks. Following that, we discuss one practical iterative usage case of the proposed model in order to provide full understanding of the whole process to the readers of this text.

**Table 7:** Resistance to network attacks (reduction in key compromise events, %).

Method	Low Attack Rate (5 %)	Medium Attack Rate (10 %)	High Attack Rate (20 %)
Proposed	12.5	15.0	18.5
[1]	8.0	10.0	12.0
[9]	9.5	12.0	15.0
[15]	9.0	11.5	14.0

## Practical use case scenario analysis

The experiment demonstrates the implementation of three components: Deep Reinforcement Learning for Adaptive QKD optimization, Variational Autoencoders for anomaly detection, and Multi-Agent Deep Q-Networks for protocol optimization. The results show efficiency, accuracy, and performance of the proposed models, with the DRL model dynamically optimizing QKD parameters under variable noise levels and channel conditions.

Iteratively, as per Table 8, it would appear that the DRL agent has successfully carried out an adjustment in the parameters of QKD under conditions of changing noise and channel loss. By using Polar Codes and LDPC error correction, the low noise at 0.01 yielded a very high secure key rate-950 kbps with a low QBER of 1.2 %. The DRL model optimizes QKD parameters in fluctuating quantum environments, reducing secure key rate and QBER. VAE, trained with normal traffic data, shows high accuracy and false positive rate.

**Table 8:** DRL for adaptive QKD optimization.

Noise Level	Channel Loss (dB)	Secure Key Rate (kbps)	QBER ( %)	Encoding Scheme	Error Correction
Low (0.01)	0.5	950	1.2	Polar Codes	LDPC
Medium (0.05)	1.0	860	2.0	Reed-Solomon	BCH
High (0.10)	2.0	720	3.8	Polar Codes	LDPC
Very High (0.15)	2.5	650	4.5	Reed-Solomon	Turbo Codes

Iteratively, as per Table 9, Performance of VAE model in quantum communication network anomaly detection. In the case of a 2 % anomaly rate, the detection accuracy was as high as 94.5 %, while the FPR was as low as 2.8 %, which implies that the model will detect the anomaly effectively with very few false alarms. When the anomaly rate was up to 20 %, the accuracy in detection decreased to 87.5 %, with a high false positive rate as high as 4.8 %. All in all, such a performance has shown that the VAE can detect such a small timing deviation of the photon transmission within the wide range of 0.002–0.020 ns with low latency in detection, which is important for real-time security in quantum network communication. Next, the MADQN approach was applied to optimize cryptographic protocols over various nodes in a distributed quantum network. Each agent chose its protocol settings autonomously based on environmental feedback: noise level, signal strength, and probability of attack. The table below describes the outcome of the protocol optimization process: key rate and resistance against attacks.

**Table 9:** VAE for anomaly detection in quantum communication networks.

Anomaly Rate ( %)	Detection Accuracy ( %)	False Positive Rate ( %)	Photon Timing Deviation (ns)	Detection Latency (ms)
2	94.5	2.8	0.002	1.2
5	92.0	3.2	0.005	1.5
10	90.2	4.0	0.010	2.0
20	87.5	4.8	0.020	2.5

Iteratively, as per Table 10, Results of protocol optimization depending on the MADQN with varied network size and attack rate. Assuming a network size of 5 nodes and an attack rate of 5 %, for instance, the optimized key rate becomes 940 kbps at QBER of 1.5 %, with an efficiency of adjustment in the protocol amounting to 20.0 %. For example, as we increased the network size and attack rates of up to 20 %, we managed to reduce the optimized key rate to 780 kbps, and the protocol adjustment efficiency is increased to 26.8 %. Attack resistance also improves significantly: under the highest attack rates, the number of key compromise events decreases by 22 % in process. These final metrics are tabulated with respect to the key rate, QBER, accuracy of anomaly detection, and levels of protocol optimization efficiencies in the following Table 11 as follows,

**Table 10:** MADQN for protocol optimization.

Network Size (Nodes)	Attack Rate ( %)	Optimized Key Rate (kbps)	QBER ( %)	Protocol Adjustment Efficiency ( %)	Attack Resistance Improvement ( %)
5	5	940	1.5	20.0	15.0
10	10	880	2.2	22.5	18.0
15	15	830	3.0	25.0	20.5
20	20	780	3.5	26.8	22.0

**Table 11:** Final outputs of the combined model.

Metric	Value
Final Secure Key Rate (kbps)	800
Final QBER ( %)	2.8
Final Anomaly Detection Accuracy ( %)	92.5
Final False Positive Rate ( %)	3.5
Final Protocol Adjustment Efficiency ( %)	25.5
Final Attack Resistance Improvement ( %)	21.5

Final outputs obtained from the combined model are listed in Table 11. Final secure key rate was 800 kbps at a QBER of 2.8 %, demonstrating efficiency in DRL-based QKD optimization. The VAE-based anomaly detection finally achieved an accuracy of 92.5 % with a false positive rate of 3.5 %. For the protocol adjustment, the model MADQN achieved 25.5 % efficiency, while the improvement in attack resistance reached 21.5 %. From these results, one can appreciate the capability of the integrated model to effectively balance major rate optimization, security enhancement, and real-time anomaly detection for large-scale quantum communication networks.

## Conclusion

The current paper discussed the advancement of quantum communication systems with state-of-the-art AI and ML techniques in a holistic approach: Deep Reinforcement Learning for adaptive QKD optimization, Variational Autoencoders for anomaly detection, Multi-Agent Deep Q-Networks for cryptographic protocol optimization. The experimental results proved that the proposed models are able to maintain high key generation rates, reduce QBER, simultaneously detect serious anomalies in real time, and fine-tune the cryptographic protocol in large quantum networks. For example, suppose the DRL-based QKD optimization scheme operates under low-noise conditions: it is able to provide a secure key rate of 950 kbps with a corresponding QBER of 1.2 %. By increasing the noise, the model is still able to generate a secure key rate of 650 kbps at QBER = 4.5 %, demonstrating the robustness of this approach in dynamic environments. This corresponds to an improvement of 15–20 % in key generation rate and a reduction of 30–40 % in QBER compared to conventional methods, confirming that DRL can cope well with fluctuations in the modelled process. BiVAE-based anomaly detection showed noteworthy results with 94.5 % accuracy in anomaly detection like eavesdropping and side-channel attacks when the anomaly rate was low, while it showed consistency with 87.5 % accuracy when the anomaly rate increased. Furthermore, the model returned an anomaly rate of only 2.8 % with regard to false positives, while a maximum anomaly rate of not >4.8 % was associated with a 25 % improvement relative to the state of the art, since this contributed to a reduction of false alarms. The MADQN framework was very effective at optimizing cryptographic protocols over many nodes in the network. In networks of 20 nodes, it is evidenced that attack resistance rose by 21.5 % in the improvement, efficiently conducted, while the improvement in attacking resistance was 25.5 %, even under high attacking conditions. Together, the approach integrated calls for quantum communication networks to meet such a delicate balance between high security standards and efficient communication under unique adversarial conditions.

## Limitations

Not Applicable.

## CRediT authorship contribution statement

**Harshala Shingne:** Conceptualization, Methodology, Software. **Diptee Chikmurge:** Visualization, Investigation. **Priya Parkhi:** Validation, Data curation, Writing – original draft. **Poorva Agrawal:** Software, Validation, Writing – review & editing.

## Declaration of competing interest

The authors declare that they have no known competing financial interests or personal relationships that could have appeared to influence the work reported in this paper.
